# ABO blood groups in relation to biomarkers of vascular structure and function in young, healthy adults – the LBA study

**DOI:** 10.1186/s12872-026-06924-0

**Published:** 2026-10-27

**Authors:** Kristina Stefansson, Gurdeep Chahal, Johan Hultdin, Torbjörn K. Nilsson, Paul Pettersson-Pablo

**Affiliations:** 1 Department of Medical Biosciences, Clinical Chemistry, Umeå University, Umeå, Sweden; 2 Department of Laboratory Medicine, Clinical Chemistry, Örebro University Hospital, Örebro, Sweden; 3 School of Medicine, Faculty of Medicine and Health, Örebro University, Örebro, Sweden

**Keywords:** ABO blood group, Cardiometabolic risk factors, Blood Glucose, Carotid intima-media thickness, Pulse-wave velocity, AIx, Healthy young subjects

## Abstract

**Background:**

In the elderly, ABO blood groups influence the risk of cardiovascular and thrombotic events, with blood group O consistently showing a more favorable risk profile. However, whether these differences affect physiological markers of vascular structure or subclinical metabolic traits in early adulthood remains poorly studied.

**Methods:**

In this cross-sectional study, cardiovascular disease-related biomarkers in serum, measures of vascular structure and function, pulse-wave velocity (PWV), augmentation index (AIx), carotid intima-media thickness (cIMT), and ABO blood groups were assessed in a sample of young, healthy subjects aged 18–26 years.

**Results:**

No statistically significant differences in PWV, AIx, or cIMT were identified across the different ABO blood groups. Conversely, the serum biomarkers showed distinct profiles. Individuals with blood group O presented with lower plasma concentrations of von Willebrand factor (vWF) compared to all non-O carriers (0.84 kIE/L vs. non-O, 1.09 kIE/L, *p* < 0.05). Furthermore, blood group O carriers demonstrated significantly lower fasting plasma glucose levels (4.96 mmol/L vs. 5.02 mmol/L, *p* = 0.02) and a trend toward lower fasting serum insulin levels (*p* = 0.07).

**Conclusion:**

ABO blood groups were not associated with measures of vascular structure and function in young, healthy adults. Non-O blood groups were associated with higher vWF levels and altered glucose metabolism, suggesting that circulating cardiometabolic risk markers vary by blood group early in life.

## Introduction

Cardiovascular diseases (CVD) constitute the leading cause of death globally among adults aged 35–70 years [1]. Although traditionally considered as conditions of older age, subclinical vascular changes develop decades before clinical events occur [2]. In the Lifestyle, Biomarkers and Atherosclerosis (LBA) study, arterial stiffness was assessed using pulse wave velocity (PWV) and augmentation index (AIx), while structural vascular changes were evaluated by carotid intima–media thickness (cIMT), all established surrogate markers of future cardiovascular risk [3–5]. Findings from the LBA study have demonstrated that cardiometabolic risk factors and vascular alterations, including subclinical atherosclerosis and increased arterial stiffness, are already evident in apparently healthy adults (18–26 years) [6, 7]. Furthermore, circulating inflammatory mediators are already detectable in this age group and seem to reflect distinct inflammatory processes, affecting different vascular beds [8].

Genetically determined traits that remain stable over the life course may be particularly valuable for identifying individuals at increased long-term cardiovascular risk at an early age. The ABO blood group system represents one such stable trait. Within the ABO blood group system, blood group O has, for a long time, at least since the 1940s, been associated with a lower risk of cardiovascular and thrombotic events [9]. This relationship has been attributed to the ABO system’s influence on plasma protein levels. Notably, low levels of von Willebrand factor (vWF) and high levels of E-selectin have been associated with blood group O, findings further supported by recent DNA methylation data [10]. Analyses of UK Biobank data (participants aged 40–69 years) have confirmed a protective association between blood group O and CVD, as well as associations between blood group O and various risk factors such as blood pressure, lipid profile, and body composition, including lower body fat and higher muscle mass [11].

Despite well-established associations between ABO blood group and cardiovascular disease (CVD) risk factors in older populations, its role in early cardiovascular risk remains incompletely understood. Given that the ABO blood group is inexpensive to determine, routinely available, and invariant over time, it may serve as an adjunctive risk marker in younger populations. The aim of this study was to examine the associations between ABO blood group and physiological biomarkers of vascular structure and function, biomarkers, and anthropometric characteristics in young, healthy adults.

## Methods

### Study population

The LBA study is a cross-sectional study evaluating healthy young adults (age range: 18–25.9 years) using previously detailed inclusion criteria [6]. The study protocol received ethical approval from the Regional Ethics Review Board in Uppsala (Ref: 2014/224). Individuals with chronic illnesses, including diabetes, subjects on medications, or a history of smoking were excluded. Participants completed two validated, computerized surveys regarding general physical health, subjective well-being, and lifestyle habits [12], with an additional question capturing self-reported hours of exercise per week. Participants selected one of three predefined categories: <2 h, 2–4 h, or > 4 h per week. Of the 834 initially recruited participants, baseline data were available for 818 individuals. Due to dropout between visits one and two of the data collection phase, insufficient sample volume, or technical limitations (e.g., insufficient ultrasound image quality), final analytical sample sizes varied slightly by parameter: ABO blood group analysis (*n* = 803), von Willebrand factor (*n* = 802), carotid intima–media thickness (*n* = 792), pulse wave velocity (*n* = 790), and augmentation index (*n* = 788). Because the percentage of missing values was relatively small, we did not perform statistical imputation.

### Dietary habits

Dietary patterns were monitored using “Matvanekollen,” a validated food frequency questionnaire developed by the Swedish National Food Agency to assess adherence to national nutritional guidelines. Although the specific digital version used in this paper no longer exists (a new, updated version was released in 2019), and was designed for broad public health use, the underlying scoring system is based on the same evaluation of individual food habits against national dietary recommendations [13]. The healthy Nordic diet represents a nutritional model centered on indigenous food sources from Northern Europe, which has demonstrated various clinical advantages. This dietary profile is primarily composed of fruit, berries, root vegetables, cabbages, legumes, fatty fish, and whole grains [14]. Test results are presented as a total score, ranging from 1 to 12. The expected score distribution was as follows: 20% (1–4 points) were considered unhealthy and encouraged to improve; 70% (5–8 points) were econsidered normal, indicating healthy habits with potential for minor enhancements; and 10% (9–12 points were considered healthy, fully aligning with the recommendations of the Swedish National Food Agency. Software-generated feedback was provided to participants based on their scores.

### Anthropometrics and physical function

Total body fat percentage was quantified by bioelectrical impedance using a Tanita BC-418 MA scale (Tanita Europe B.V., Amsterdam, The Netherlands). Handgrip strength (HGS) was measured using the dominant hand with a HiRes hydraulic hand dynamometer (Fabrication Enterprises Inc., Irvington, NY). Following an initial trial run, the final HGS value was recorded as the mean of three consecutive trials.

### Vascular structure and function

Vascular assessments encompassed both functional and structural metrics, utilizing average values from triplicate readings taken on a single day, as described previously in detail [15]. We measured pulse wave velocity (PWV) and augmentation index (AIx) using applanation tonometry (SphygmoCor, AtCor Medical Pty Ltd, Sydney, Australia). For PWV, carotid and femoral arterial pulse waves were recorded simultaneously with electrocardiography to determine the pulse transit time. Distance was measured as a straight line from the sternal notch to the carotid site and from the sternal notch to the femoral site via the umbilicus. The carotid-to-sternal distance was subtracted from the femoral-to-sternal distance to calculate path length. For AIx, we performed radial artery tonometry on the right wrist. The central aortic pressure waveform was derived using the built-in, validated transfer function of the SphygmoCor system, with values standardized to a reference heart rate of 75 beats per minute [16]. Carotid-intima media thickness (cIMT) was imaged via a high-resolution ultrasound B-mode system (GE Healthcare, Vivid E9, Chicago, IL, USA) equipped with a 12 MHz linear array transducer.

### Laboratory analyses

Blood samples were drawn into vacuum tubes (BD Vacutainer; BD AB, Stockholm, Sweden) following an overnight fast. Sodium citrate-fluoride Vacutainer tubes were used for glucose determination. Serum and plasma were collected in lithium-heparin Vacutainer tubes for the remaining analyses. C-reactive protein (CRP) was measured with a high-sensitivity method on a Siemens ADVIA 1800 Chemistry instrument using the Siemens hsCRP Assay (Siemens Healthineers, Upplands Väsby, Sweden). Fasting glucose and lipid profiles, including total cholesterol (CHOL), triglycerides (TG), and high-density lipoprotein (HDL), were determined colorimetrically with Vitros MicroSlide technology. Direct low-density lipoprotein (LDL) was analyzed using a two-step colorimetric assay with Vitros MicroWell technology, on a Vitros 5.1 system (Clinical Chemistry Instruments, Raritan, NJ, USA). Creatinine and cystatin C were measured on a Siemens ADVIA Chemistry XPT platform, and homocysteine and vitamins B12 and folate were quantified using a Siemens ADVIA Centaur XPT system (Siemens Healthineers).

### ABO blood grouping

For blood group analysis, reverse blood grouping was performed using gel cards (NaCl, Enzyme Test and Cold Agglutinins (Id-n°: 50520; DiaMed GmbH, Cressier, Switzerland**)**, with test erythrocytes from Laboratory Medicine, Clinical Immunology and Transfusion Medicine (Umeå University Hospital, Umeå, Sweden). Plasma samples were centrifuged at 10,000 g for 10 min to remove fibrin strands and prevent incorrect grouping. The analysis was performed according to the manufacturer’s instructions, except that the sample plasma volume was reduced from 50 µL to 25 µL per well. This modified volume was validated prior to study initiation in our laboratory using both 25 µL and 50 µL plasma volumes, demonstrating comparable signal intensities and assay performance. This adapted protocol has also been successfully applied in previous studies [17]. Internal quality control samples representing blood groups O, A, and AB were run routinely throughout all analytical batches and consistently yielded the expected results. The gel cards were subsequently analyzed, and reactivity with test cells was graded successfully for ABO blood group in 803 plasma samples, whereas in 15 samples ABO blood group could not be determined due to technical reasons.

### Statistics

All statistical analyses were performed in SPSS, v. 29 (IBM, Armonk, NY, USA). Normal distribution was assessed by comparing the SD to the mean, and graphically displayed in a histogram for visual evaluation. For skewed distributions, a natural logarithmic transformation was employed. Baseline characteristics in Table 1 are presented by sex to evaluate cohort normalcy and illustrate the expected physiological sex differences, which were compared using independent-samples t-tests. Subsequently, sex-specific z-score transformations were applied to all continuous variables prior to pooling the cohort for further analyses. Differences between the four blood groups were analyzed using one-way analysis of variance (ANOVA). To compare the O group against the non-O group, independent samples t-test was performed. Primary outcomes were defined a priori as the three structural and functional vascular parameters (PWV, AIx, and cIMT). Secondary analyses of metabolic, laboratory, and anthropometric parameters were exploratory and hypothesis-generating; therefore, to minimize the risk of Type II errors (false negatives), no adjustments for multiple comparisons were applied across secondary endpoints. Statistical significance was set at *p* < 0.05.

**Table 1:** – Baseline characteristics (mean ± SD) for young men and women of the LBA cohort

Parameters	Men	Women	*p* value
n	253	565	
Age, years	22 ± 2.0	22 ± 1.9	0.23
*Anthropometry and physical fitness*			
Height, cm	182 ± 6.70	169 ± 6.33	< 0.001
Weight, kg	77 ± 11	64 ± 11	< 0.001
Fat-free mass, kg	66 ± 8.0	45 ± 4.4	< 0.001
BMI, kg/m^2^	23 ± 3.1	22 ± 3.6	< 0.001
Body fat, %	15 ± 5.6	28 ± 6.6	< 0.001
Waist circumference, cm	82 ± 7.3	74 ± 7.9	< 0.001
Oxygen uptake (VO_2_), mL/min/kg	3.3 ± 0.86	2.4 ± 0.54	< 0.001
Hand grip strength, kg	53 ± 10	34 ± 6.5	< 0.001
*Biochemical variables*			
Total cholesterol, mmol/L	4.05 ± 0.79	4.31 ± 0.77	< 0.001
LDL-cholesterol, mmol/L	2.30 ± 0.68	2.3 ± 0.71	0.80
HDL-cholesterol, mmol/L	1.24 ± 0.28	1.45 ± 0.36	< 0.001
Triglycerides, mmol/L	0.79 ± 0.35	0.81 ± 0.37	0.40
Lp(a), nmol/L	31.4 ± 59	39.1 ± 60	0.089
vWF, kIE/L	1.02 ± 0.44	0.98 ± 0.43	0.21
CRP, mg/L	1.3 ± 2.7	1.9 ± 4.0	0.027
Fasting serum insulin, mIE/L	7.5 ± 3.7	8.0 ± 4.5	0.14
Fasting plasma glucose, mmol/L	5.2 ± 0.32	4.9 ± 0.34	< 0.001
Vitamin B12, pmol/L	345 ± 150	304 ± 108	< 0.001
Folate, nmol/L	16 ± 8.5	17 ± 10	0.012
Homocysteine, µmol/L	14 ± 6.0	12 ± 3.5	< 0.001
*Lifestyle variables*			
Hours of exercise/week, h	2.2 ± 0.97	2.2 ± 0.88	0.33
Food habits score	5.8 ± 1.9	6.1 ± 1.9	0.062
*Vascular measurements*			
Systolic blood pressure, mmHg	124 ± 12	111 ± 9.1	< 0.001
Average MVPA/day, min	50 ± 26	53 ± 23	0.12
PWV, m/s	5.6 ± 0.86	5.2 ± 0.72	< 0.001
AIx	-8.5 ± 9.5	-5.0 ± 9.9	< 0.001
cIMT, mm	0.50 ± 0.064	0.49 ± 0.057	0.030

Abbreviations: BMI, body mass index; Lp(a), lipoprotein (a); vWF, von Willebrand factor; CRP, C-reactive protein; MVPA, moderate and vigorous physical activity; PWV, pulse wave velocity; AIx, augmentation index; cIMT, carotid intima–media thickness

## Results

Table 1 presents baseline demographics, lifestyle behaviors, anthropometrics, and physiological profiles by sex. A total of 818 young, healthy adults were evaluated, 253 men (31%) and 565 women (69%), with a mean age of 22 years for both sexes. Several statistically significant mean differences between sexes were observed across several parameters, notably anthropometrics, as expected. For laboratory variables, women had higher total cholesterol and HDL cholesterol, whereas men had higher plasma glucose, vitamin B12, and homocysteine. Vascular measurements also differed by sex, with men presenting higher systolic blood pressure and pulse wave velocity (PWV), while women exhibited a less negative mean augmentation index (AIx). Mean carotid intima-media thickness (cIMT) was similar between sexes.

All measured parameters were evaluated across the four ABO blood groups, as well as compared between group O and non-O carriers (Table 2). Vascular measurements showed no statistically significant differences across ABO blood groups or between O and non-O groups, but a lower systolic blood pressure was observed in O than in non-O that was close to significant (*p* = 0.077). PWV and AIx were nearly identical among all groups. cIMT was very slightly lower in the AB group than in the other blood groups (whose average thickness of 0.50 mm matched the population average of 0.50 mm). Similarly, no significant differences were observed for anthropometric metrics, lipid profiles, inflammation markers, blood pressure, or lifestyle variables. However, among lifestyle variables, reported exercise hours per week showed a trend toward significance across the four blood groups (*p* = 0.077, with group AB reporting fewer exercise hours), alongside a trend for daily MVPA when comparing O and non-O groups (*p* = 0.075).

**Table 2:** – Measurements according to ABO blood group (mean ± SD)

Parameters	A	B	AB	O	p*	Non-O	O	p*
n (%)	375 (46.70%)	93 (11.58%)	35 (4.36%)	300 (37.36%)		503 (62.64%)	300 (37.36%)	
Age, years	21.88 ± 1.97	21.75 ± 2.04	22.11 ± 1.92	21.93 ± 1.90	0.79	21.87 ± 1.97	21.93 ± 1.90	0.69
*Anthropometry and physical fitness*								
Height, cm	172.69 ± 9.02	171.58 ± 9.60	172.49 ± 9.08	173.00 ± 8.57	0.62	172.47 ± 9.13	173.00 ± 8.57	0.42
Weight, kg	68.22 ± 12.68	67.83 ± 13.33	66.31 ± 10.41	68.28 ± 13.81	0.85	68.01 ± 12.64	68.28 ± 13.81	0.78
Fat-free mass, kg	65.69 ± 7.98	66.79 ± 6.48	65.63 ± 7.81	65.60 ± 8.67	0.91	65.89 ± 7.67	65.60 ± 8.67	0.78
BMI, kg/m2	22.79 ± 3.39	22.93 ± 3.57	22.21 ± 2.60	22.72 ± 3.70	0.77	22.78 ± 3.37	22.72 ± 3.70	0.82
Body fat, %	24.05 ± 8.76	23.67 ± 8.85	24.09 ± 9.18	23.90 ± 8.85	0.98	23.98 ± 8.79	23.90 ± 8.85	0.90
Waist circumference, cm	76.50 ± 8.36	76.19 ± 8.17	76.06 ± 7.47	76.41 ± 9.13	0.98	76.41 ± 8.26	76.41 ± 9.13	0.99
Oxygen uptake (VO2), mL/min/kg	39.33 ± 9.37	38.86 ± 8.72	38.32 ± 7.97	39.83 ± 9.42	0.70	39.18 ± 9.15	39.83 ± 9.42	0.34
Hand grip strength, kg	40.39 ± 11.72	40.88 ± 12.55	41.50 ± 13.34	39.98 ± 11.44	0.85	40.55 ± 11.97	39.98 ± 11.44	0.51
*Biochemical variables*								
Total cholesterol, mmol/L	4.23 ± 0.79	4.33 ± 0.95	4.14 ± 0.82	4.18 ± 0.72	0.42	4.24 ± 0.82	4.18 ± 0.72	0.29
LDL-cholesterol, mmol/L	2.32 ± 0.70	2.38 ± 0.85	2.34 ± 0.73	2.27 ± 0.67	0.53	2.33 ± 0.73	2.27 ± 0.67	0.20
HDL-cholesterol, mmol/L	1.38 ± 0.37	1.39 ± 0.37	1.26 ± 0.29	1.40 ± 0.34	0.19	1.37 ± 0.36	1.40 ± 0.34	0.37
Triglycerides, mmol/L	0.81 ± 0.34	0.84 ± 0.49	0.81 ± 0.35	0.80 ± 0.35	0.74	0.81 ± 0.37	0.80 ± 0.35	0.50
Lp(a), nmol/L	34.13 ± 54.25	33.65 ± 50.39	33.02 ± 44.76	41.47 ± 69.50	0.39	33.96 ± 52.85	41.47 ± 69.50	0.085
vWF, kIE/L	1.09 ± 0.46	1.07 ± 0.47	1.20 ± 0.56	0.84 ± 0.32	< 0.001	1.09 ± 0.47	0.84 ± 0.32	< 0.001
CRP, mg/L	1.71 ± 3.90	1.22 ± 1.70	0.98 ± 1.09	1.99 ± 4.04	0.20	1.57 ± 3.46	1.99 ± 4.04	0.12
Fasting serum insulin, mIE/L	8.01 ± 4.92	8.13 ± 3.62	7.93 ± 3.17	7.46 ± 3.79	0.35	8.03 ± 4.59	7.46 ± 3.79	0.074
Fasting plasma glucose, mmol/L	5.03 ± 0.35	5.04 ± 0.34	4.96 ± 0.37	4.96 ± 0.35	0.084	5.02 ± 0.35	4.96 ± 0.35	0.020
Vitamin B12, pmol/L	321 ± 133	329 ± 123	319 ± 98	307 ± 115	0.34	322 ± 129	307 ± 115	0.083
Folate, nmol/L	16.53 ± 8.44	17.88 ± 8.54	15.84 ± 9.54	16.99 ± 11.54	0.62	16.73 ± 8.54	16.99 ± 11.54	0.72
Homocysteine, µmol/L	12.59 ± 4.19	12.32 ± 4.31	11.98 ± 3.93	12.66 ± 5.22	0.81	12.49 ± 4.19	12.66 ± 5.22	0.62
*Lifestyle variables*								
Hours of exercise/week, h	2.18 ± 0.88	2.26 ± 0.85	1.80 ± 1.16	2.19 ± 0.93	0.077	2.17 ± 0.90	2.19 ± 0.93	0.69
Average MVPA/day, min	50.95 ± 21.57	51.99 ± 24.23	46.64 ± 21.67	53.99 ± 25.98	0.22	50.84 ± 22.07	53.99 ± 25.98	0.075
Food habits score	5.98 ± 1.91	5.99 ± 1.79	5.71 ± 1.71	6.02 ± 1.89	0.84	5.96 ± 1.88	6.02 ± 1.89	0.68
*Vascular measurements*								
Systolic blood pressure, mmHg	115.64 ± 12.22	116.34 ± 11.94	115.20 ± 12.98	114.20 ± 11.51	0.33	115.74 ± 12.20	114.20 ± 11.51	0.077
PWV, m/s	5.33 ± 0.76	5.31 ± 0.81	5.30 ± 0.88	5.33 ± 0.77	0.99	5.32 ± 0.78	5.33 ± 0.77	0.87
AIx	-6.36 ± 9.56	-5.37 ± 9.63	-6.11 ± 10.00	-6.10 ± 10.39	0.86	-6.16 ± 9.59	-6.10 ± 10.39	0.94
cIMT, mm	0.49 ± 0.059	0.50 ± 0.052	0.48 ± 0.066	0.50 ± 0.061	0.34	0.49 ± 0.059	0.50 ± 0.061	0.17

Abbreviations: BMI, body mass index; Lp(a), lipoprotein (a); vWF, von Willebrand factor; CRP, C-reactive protein; MVPA, moderate and vigorous physical activity; PWV, pulse wave velocity; AIx, augmentation index; cIMT, carotid intima–media thickness. To obtain p-values with ANOVA for all subjects, data were z-scored for men and women separately and then merged

In contrast, plasma von Willebrand factor (vWF) concentrations differed significantly across blood groups (*p* < 0.001), with individuals in blood group O having lower vWF levels (0.84 kIE/L) than non-O carriers (mean, 1.09 kIE/L). Additionally, blood group O carriers had significantly lower fasting plasma glucose levels than non-O carriers (4.96 mmol/L vs. 5.02 mmol/L, *p* = 0.020) and a trend toward lower fasting serum insulin (7.46 mIE/L vs. 8.03 mIE/L, *p* = 0.074, two-sided test) (Table 2).

## Discussion

In this study, we investigated various physiological and biochemical CVD risk markers in a young, healthy population, comparing them by ABO blood group. We found no association between ABO blood groups and vascular structure and function parameters in this age bracket. Still, blood group O had a non-statistically significant lower systolic blood pressure than non-O (114.20 mmHg vs. 115.74 mmHg, *p* = 0.077). Large-scale genome-wide association studies have identified links between genetic loci, such as methyl-tetrahydrofolate, MTHFR, and blood pressure regulation as well as hypertension susceptibility in large cohorts [18]. While blood pressure did not differ significantly, the tendency towards a lower systolic blood pressure in blood group O in our young, healthy cohort could suggest that genetic variations require additional factors to overtly manifest, such as interactions with age, prolonged cumulative exposure, or other synergistic risk factors, to manifest as distinct clinical differences. Associations between ABO blood groups and vascular structure remain uncertain, with some clinical studies in the elderly having found differences between blood group O and non-O individuals: Blood group A has previously been associated with higher coronary artery calcium scores (CACS) and more severe arterial stenosis, and blood group O with lower CACS and more stable plaques [19, 20]. In a study of healthy, middle-aged individuals, no significant association was observed between ABO blood group (O vs. non-O) and subclinical atherosclerosis, as assessed by cIMT and carotid plaques [21]. This accords with our findings in our younger cohort, which in addition included measures of arterial stiffness (PWV and AIx). The absence of macrovascular changes in our young cohort indicates that the overt protective phenotype of blood group O against clinical events does not originate from early-adult developmental structural features of the large-vessel walls. Instead, macrovascular differences reported in older populations may reflect the cumulative impact of metabolic or inflammatory exposures over time rather than primary structural variations present in early adulthood.

Although vascular alterations were still absent, we confirmed significant differences in vWF between O and non-O individuals (Table 2). The lower mean vWF concentration in group O participants aligns with the ABO locus directly modulating the glycosylation and clearance rates of circulating vWF, leading to lower baseline levels in O carriers [9, 10]. Given vWF’s dual role of in platelet adhesion and as a carrier for coagulation Factor VIII, the lower systemic levels in blood group O carriers confer a persistent anti-thrombotic advantage from early adulthood onward.

Of perhaps even higher interest is our finding that blood group O had lower fasting glucose and insulin than non-O blood groups (Table 2). Larger studies and meta-analyses indicate that individuals with non-blood type O have a higher risk of diabetes mellitus type 2 [22, 23] and of developing gestational diabetes mellitus [24]. However, the direct relationship between ABO antigens and subclinical glucose regulation in healthy cohorts has historically yielded conflicting results, particularly in obstetric or metabolic sub-populations where blood group O has been paradoxically associated with both higher and lower plasma glucose [25–27]. Our findings in this young, non-pregnant, disease-free cohort support a baseline metabolic benefit for blood group O. The lower fasting glucose and modest reductions in insulin in O carriers suggest that non-O carriers might maintain slightly higher subclinical insulin resistance early in adult life, which could accelerate long-term cardiovascular risks. This is consistent with recent publications suggesting that non-O blood types are a risk factor for the development of diabetes [22, 28]. Given that the expression of ABO blood group antigens in the intestinal mucus is dictated by specific patterns of mucosal glycosylation, it has been suggested that this relationship may be mediated in part by an interplay between ABO blood groups and variations in the gut microbiome [29, 30]. Furthermore, lifestyle factors should be considered when interpreting these early metabolic differences. Although not statistically significant, weekly exercise hours showed a trend across blood groups (*p* = 0.077), with the AB group reporting lower exercise duration, as did daily MVPA between O and non-O groups (*p* = 0.075). Whether genetic variation at the ABO locus could subtly influence physiological exercise tolerance or athletic performance remains an area of exploratory interest in sports physiology [31], though current evidence supporting direct ABO-driven behavioral exercise propensity remains limited. More likely, given that physical activity directly influences insulin sensitivity and glucose handling [32], subtle variations in exercise habits across ABO phenotypes could act as a contributing factor in these early cardiometabolic marker differences.

There are limitations to consider when interpreting our findings. Due to the cross-sectional design of the LBA study, we cannot establish direct causality regarding the influence of ABO blood groups on early metabolic pathways. Second, our cohort exhibits a notable sex imbalance. Although sex-specific *z*-score transformations were used to account for baseline physiological differences prior to pooled analyses, the smaller proportion of male participants reduced the statistical power for detecting potential sex-specific subgroup differences or associations. As for our missing data, we performed no imputation; we had a complete-case approach to the statistical analyses. We considered the missing data minor, but varying sample sizes across sub-analyses remain a limitation. Furthermore, the ABO blood grouping was determined by reverse blood grouping of plasma samples. The results may differ from those obtained with a typing protocol based on erythrocyte antigen testing in sporadic cases. Finally, differences in methods to assess the vascular system in the previous studies have to be taken into account: the early studies, e.g., Kingsbury, relied on the clinical diagnostic tools available at the time [33], whereas more recent studies (including the present one) utilize advanced imaging methods or physiological measurements of the vascular tree. The different methods may vary in their ability to detect the earliest vascular changes associated with the ABO system.

In conclusion, we demonstrate that young, healthy adults aged 18–26 years do not exhibit any ABO-dependent changes in physiological measures of vascular structure and function. This contrasts with what has been repeatedly documented in middle-aged adults and seniors with manifest cardiovascular disease, but it aligns with previous reports on middle-aged healthy volunteers. In our present cohort, non-O blood groups are associated with higher vWF levels and higher fasting plasma glucose. We speculate that the mechanism(s) by which non-O blood groups mediate a higher long-term cardiovascular burden are driven early on by higher baseline vWF concentrations and differences in glucose metabolism. These are potential pathways through which ABO blood groups may influence cardiovascular risk profiles prior to the onset of measurable vascular change.

## Data Availability

The data or part of the data that support the findings of this study are available upon reasonable request from the corresponding author, PPP. The data are not publicly available due to their containing information that could compromise the privacy of research participants.

## Abbreviations

AIx: augmentation index
BMI: body mass index
CACS: coronary artery calcium scores
cIMT: carotid intima-media thickness
CRP: C-reactive protein
CVD: cardiovascular disease
HDL: high-density lipoprotein
LBA: the lifestyle, biomarkers and atherosclerosis study
LDL: low-density lipoprotein
Lp(a): lipoprotein (a)
MVPA: moderate and vigorous physical activity
PWV: pulse-wave velocity
TG: triglyceride
vWF: von Willebrand factor
